# The lung mycobiome: an emerging field of the human respiratory microbiome

**DOI:** 10.3389/fmicb.2015.00089

**Published:** 2015-02-13

**Authors:** Linh D. N. Nguyen, Eric Viscogliosi, Laurence Delhaes

**Affiliations:** ^1^Biology and Diversity of Emerging Eukaryotic Pathogens, Center for Infection and Immunity of Lille, INSERM U1019, CNRS UMR 8204, Lille Pasteur Institute, University of Lille Nord de France, Lille, France; ^2^Parasitology-Mycology Department, Hospital University Center, Faculty of Medicine, Lille, France

**Keywords:** respiratory mycobiome, mycobiota, fungal microbiota, microbiome, lung, chronic respiratory disease

## Abstract

The lung microbiome, which is believed to be stable or at least transient in healthy people, is now considered as a poly-microorganism component contributing to disease pathogenesis. Most research studies on the respiratory microbiome have focused on bacteria and their impact on lung health, but there is evidence that other non-bacterial organisms, comprising the viruses (virome) and fungi (mycobiome), are also likely to play an important role in healthy people as well as in patients. In the last few years, the lung mycobiome (previously named the fungal microbiota or microbiome) has drawn closer attention. There is growing evidence that the lung mycobiome has a significant impact on clinical outcome of chronic respiratory diseases (CRD) such as asthma, chronic obstructive pulmonary disease, cystic fibrosis, and bronchiectasis. Thanks to advances in culture independent methods, especially next generation sequencing, a number of fungi not detected by culture methods have been molecularly identified in human lungs. It has been shown that the structure and diversity of the lung mycobiome vary in different populations (healthy and different diseased individuals) which could play a role in CRD. Moreover, the link between lung mycobiome and different biomes of other body sites, especially the gut, has also been unraveled. By interacting with the bacteriome and/or virome, the respiratory mycobiome appears to be a cofactor in inflammation and in the host immune response, and therefore may contribute to the decline of the lung function and the disease progression. In this review, we report the recent limited explorations of the human respiratory mycobiome, and discuss the mycobiome’s connections with other local microbial communities, as well as the relationships with the different biomes of other body sites. These studies suggest several outlooks for this understudied emerging field, which will certainly call for a renewal of our understanding of pulmonary diseases.

## INTRODUCTION

The recent use of culture-independent microbiological techniques based on deep-sequencing has shown that the respiratory tract of healthy people are not sterile as formerly thought, but composed of a previously unappreciated complex microbial community referred as the microbiome (Charlson et al., 2010; Erb-Downward et al., 2011). In particular, two international projects were recently launched: the American Human Microbiome Project (HMP)^1^ and the European Metagenomics of the Human Intestinal Tract (MetaHit^2^; The NIH HMP Working Group et al., 2009; Huttenhower et al., 2012). They first aimed to characterize the nature, composition, and diversity of normal and pathological bacteriomes (bacterial microbiomes) at different body sites including the nasal passages, oral cavities, skin, gastrointestinal tract, and urogenital tract. The respiratory tract was secondarily included in the American program as a body site, and the lung microbiome has become a new attractive and rapidly growing field of research. However, the majority of these lung microbiome studies have focused on bacteria; the characterization of viral or fungal microbiomes—also referred as the virome and mycobiome—has not been closely investigated. It is commonly accepted that a given microbial community associated with host tissues or organs is known as the “biota,” while the whole genome collection of this microbial community is mentioned as the “biome.” Accordingly, “mycobiota” refers to the fungal component of a given microbial community, and the corresponding genomes are referred to as the “mycobiome” (Hooper, 2001; Iliev et al., 2012; Orgiazzi et al., 2013).

While the concept of the human mycobiome has been emphasized in the past few years (Cui et al., 2013; Huffnagle and Noverr, 2013), little is known about the human mycobiome in general, and the lung mycobiome in particular as confirmed by our search in PubMed of the terms: “mycobiota,” “mycobiome,” “human mycobiota,” “human mycobiome,” “mycobiome AND lungs,” “mycobiota AND lungs,” “human mycobiome AND lungs,” “human microbiota and lungs,” “fungal microbiome AND lungs” that appeared respectively in 190, 29, 49, 24, 272, 1, 188, 213, and 146 publications. With or without using these specific keywords, a total of 67 research publications and 29 reviews were relevant to the purpose of our review. Lung mycobiome research is clearly an “emerging world” as recently proposed (Huffnagle and Noverr, 2013), for which the growing scientific interest is supported by several reasons.

First, the human respiratory tract represents the main portal of entry for numerous microorganisms primarily those occurring as airborne particles such as viruses, bacteria, or fungi. Fungal spores are representing more than 50,000 spores per cubic meter of air during the fungal season (Pashley et al., 2012; Denning et al., 2014). The corresponding microorganism characteristics, coupled with the local immune response will determine whether they will be cleared or adhere to and colonize the respiratory tract, leading to pulmonary diseases (or their infectious complications). Although the total number of fungal cells is smaller than that of the bacteria, nobody is fungus-free (Huffnagle and Noverr, 2013).

Second, mycosis represents an emergent threat to public health, which has increased not only in width, but also in depth. Whereas fungal pulmonary infections represent life-threatening diseases in patients in a comparable rate to the mortality attributable to tuberculosis or malaria, it is still difficult to diagnose and treat them. Beside the most prevalent and well-known fungal pathogens such as *Candida albicans* and *Aspergillus fumigatus*, a large number of new emerging pathogens have been described (Horré et al., 2010; Fisher et al., 2012; Marguet et al., 2012). In addition, the widespread use of antibiotics has probably influenced the balance between bacterial and fungal infections facilitating the occurrence of respiratory mycosis that are becoming resistant to antifungal drugs and difficult to treat (Chen et al., 2012; Vermeulen et al., 2013; Bousquet et al., 2014).

Third, common links exist between fungi and diseases such as chronic respiratory diseases (CRD) through the immunogenic background of each individual. As part of the continuous host–pathogen–environment interaction from birth to death, fungi have been associated with asthma, chronic obstructive pulmonary disease (COPD), cystic fibrosis (CF), bronchiectasis at different levels (such as fungal isolation in a sputum sample, sensitization to *A. fumigatus* or to other fungi; Fillaux et al., 2012; Knutsen et al., 2012; Speirs et al., 2012; Armstead et al., 2014; Denning et al., 2014). The presence of fungi in respiratory tract also related to worse CRD outcome (Amin et al., 2010; Chotirmall et al., 2010).

Despite the fact that knowledge of the mycobiome lags behind our understanding of the bacterial microbiome, specific mycobiota have been identified in pulmonary diseases as well as in oral, digestive, and skin diseases (Ghannoum et al., 2010; Lu et al., 2011; Charlson et al., 2012b; Delhaes et al., 2012; Knutsen et al., 2012; Harrison et al., 2013; Huang et al., 2013; Dupuy et al., 2014; Willger et al., 2014). Currently, the abundance of fungi in different body sites seems to be many degrees smaller than bacteriome as recently reported (Marsland and Gollwitzer, 2014; Underhill and Iliev, 2014). For example, gut mycobiome has been evaluated at less than 0.1% in human feces upon the MetaHIT project (Huffnagle and Noverr, 2013). Since the development of the host immune system depends on its symbiotic relationship with the microbiome, the mycobiome may act as a cofactor in the lung inflammatory response (Marsland and Gollwitzer, 2014). Therefore, it appears to be essential to understand more about respiratory mycobiome, and its potential interaction with other biomes in order to get a complete picture of the lung microbiome and to improve patient management by changing current paradigms of the disease.

In this review, we summarize the recent advances in characterizing the respiratory mycobiome of patients with CRD, and discuss the mycobiome’s connections with other local microbial communities, as well as the relationships with the different biomes of other body sites. These studies suggest several outlooks for this emerging field which will certainly call for a renewal of our understanding of pulmonary diseases.

## RESPIRATORY MYCOBIOME: FROM PROOF OF CONCEPT TO RATIONAL DATA

Conventional microbial methods such as cultures of respiratory samples are useful in diagnosing fungal infections and to isolating and phenotyping microorganisms. However, they have some limitations in identifying co-infections and dynamics of polymicrobial populations. These communities consist of bacteria, fungi, and viruses all potentially contributing to infection and inflammation (Lu et al., 2011; Charlson et al., 2012a,b; Delhaes et al., 2012; Fodor et al., 2012; Filkins et al., 2012; Carmody et al., 2013; Cui et al., 2013; Harrison et al., 2013; Huang et al., 2013; Dawood et al., 2014; Fitzpatrick et al., 2014; Goffard et al., 2014; Lim et al., 2014; McCullers, 2014; Mounier et al., 2014; Purcell et al., 2014; Willger et al., 2014; Wurzel et al., 2014). In addition, the majority of fungal species are difficult to cultivate or uncultivable on usual culture media as well as new or unknown pathogens (Bittar et al., 2008; Chabé et al., 2011; Paniz-Mondolfi et al., 2012). In this context, deep-sequencing methods provide an indisputable possibility to identify all microbial species (including those difficult to culture) and to generate exhaustive microbial community data. Despite recent efforts to develop high-throughput methods targeting fungal ribosomal RNA genes to characterize fungal microbiota (recently summarized by Cui et al. (2013), the published data have mainly explored the lung bacteriome. Only few studies have addressed respiratory community and diversity of fungi (Charlson et al., 2012a,b; Delhaes et al., 2012; Harrison et al., 2013; van Woerden et al., 2013; Willger et al., 2014) or viruses and phages (Willner et al., 2011, 2012; Lim et al., 2013). But there is growing evidence to suggest the impact of viral infection on pulmonary exacerbation, and mold-related respiratory effects on CRD (Reponen et al., 2011, 2012; Hulin et al., 2013; Kieninger et al., 2013).

Briefly, each lung microbiota (including mycobiota) has its own composition and evolution, which is unique and specific to each individual. It probably evolves according to the pulmonary disease and to the occurrence or not of an acute pulmonary exacerbation (Fodor et al., 2012; Carmody et al., 2013; Lim et al., 2014; Willger et al., 2014). As a consequence it is believed to play a role in the alteration of the lung function.

**FIGURE 1:**
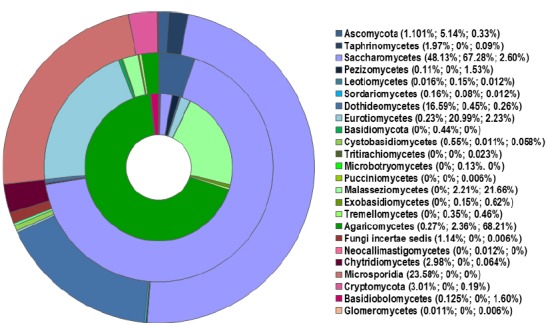
**Distribution of fungal classes (in % of relative abundance) in the sputum of healthy individuals (outer ring) and patients with CF (middle ring) and asthma (inner ring), based on published pyrosequencing investigations (Delhaes et al., 2012; van Woerden et al., 2013).** The percentages on the legend correspond to each class identified in healthy, CF, and asthma populations (from the outer to inner rings respectively). Reads that were not identified as class level are group at phylum levels (Ascomycota, Basidiomycota). Classes less than 0.1% are not represented in the rings; the class named “Fungi incertae sedis” refers to unclassified fungi.

**FIGURE 2:**
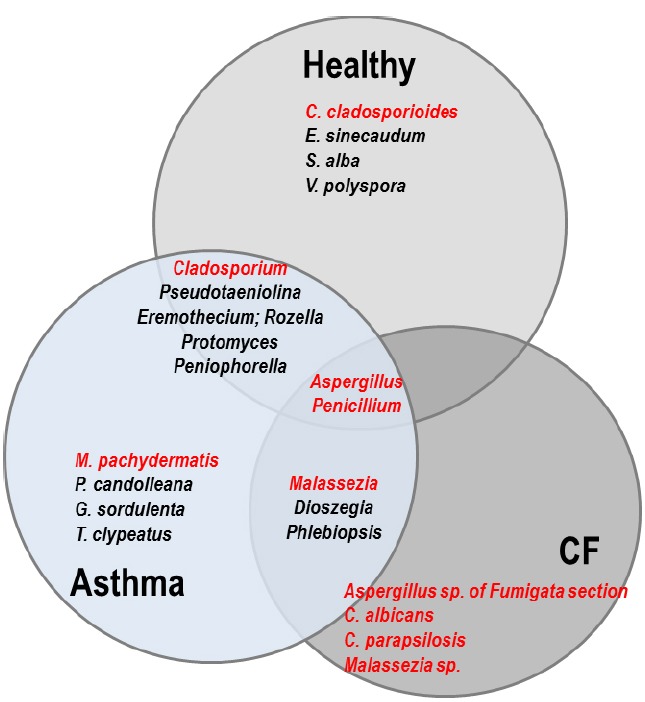
**Venn diagram representing the comparison of the respiratory mycobiomes in healthy individuals, and patients with CF or asthma from published studies (Delhaes et al., 2012; van Woerden et al., 2013; Willger et al., 2014).** Shared genera are indicated in the overlap regions. The four most frequent species specifically isolated in each population are indicated in the non-overlap regions. Genera and species in red represent known opportunistic pathogens. In healthy people, *E. sinecaudum, Vanderwaltozyma polyspora*, and *Systenostrema alba* are Saccharomycetaceae and microsporidia isolated from soil and plants with no known clinical pathogenicity. *Cladosporium cladosporioides* has been described in cutaneous, subcutaneous, lung, and disseminated infections in immunocompromised patients. In the asthma population, *Psathyrella candolleana, Grifola sordulenta*, and *Termitomyces clypeatus* are environnemental Basidomycota. *Malassezia pachydermatis* is associated with atopic dermatitis. In CF patients, exclusively known opportunistic pathogens are the most frequent: *Aspergillus* species belonging to the Fumigata section are responsible for allergic disease (ABPA) as well as infection, while the pathogenicity of yeasts (*Candida* and *Malassezia*) is still a matter of debate in the context of CF. Among the shared fungal communities: *Eremothecium* is a filamentous fungus originally isolated from cotton; *Pseudotaeniolina* members are environmental fungi and occur only rarely in human hosts; *Rozella* is a widespread genus considered one of the earliest diverging lineages of fungi, isolated from the environment (marine); *Protomyces* is an Ascomycota phytopathogen; *Peniophorella* are soil Basidiomycota, of which few species are restricted to the northern hemisphere; *Dioszegia* are basidiomycetous yeasts found in a wide range of habitats; *Phlebiopsis* are saprotrophic fungi with a widespread distribution. *Cladosporium* species are becoming increasingly important opportunistic pathogens, especially in solid organ transplant recipients. *Malassezia* are members of the human skin flora which are associated with a wide spectrum of clinical manifestations from benign skin conditions (tinea versicolor) to fungemia in the immunocompromised host, or atopic dermatitis.

The lung mycobiome of healthy people are comprised various genus and species principally dominated by environment agents including *Aspergillus* species (Figures 1 and 2; Charlson et al., 2012b; van Woerden et al., 2013; Marsland and Gollwitzer, 2014; Underhill and Iliev, 2014). For example, in the study of Charlson et al. (2012b), the most common taxa in healthy control groups are *Davidiellaceae*, *Cladosporium*, *Eurotium*, *Penicillium.* Similarly, the most abundant species of van Woerden’s study (van Woerden et al., 2013) were environmental molds normally isolated from water, plant, or soil samples such as *Cladosporium cladosporioides* and *Eremothecium sinecaudum*.

The lung mycobiota of diseased subjects has been analyzed using deep-sequencing approaches for the first time in two studies conducted in 2012 (Charlson et al., 2012b; Delhaes et al., 2012). Delhaes et al. (2012) have studied the sputum samples of CF patients while Charlson et al. (2012b) investigated the bronchoalveolar lavage (BAL) and oropharyngeal wash (OW) samples of lung transplant patients suffering previously from different diseases such as CF, COPD, idiopathic pulmonary fibrosis, interstitial lung disease, and cardiovascular disease. In spite of some differences in the methodology of DNA extractions and data analysis between the two studies, the most common species or genera found in the lung mycobiome were: *C. albicans*, *Aspergillus* spp*.*, *Penicillium*, *Cryptococcus, Eurotium*, in which *Candida* species dominated (Figure 1). It was reported that more than 60% of the species or genera of the fungal communities identified using 454 FLX technology (Life Sciences) were not detected by cultures (Delhaes et al., 2012). These results were recently confirmed (Willger et al., 2014). Charlson et al. (2012b) also applied the pyrosequencing method to characterize the airway microbiome in both the lower and upper respiratory tracts of lung transplant patients. It was shown that the respiratory microbial communities in lung transplant recipients differ in structure and composition from those of healthy subjects which were found to be similar to the oral microbiome described in another recent study (Ghannoum et al., 2010). The lower respiratory tract of lung transplant subjects exhibited higher burdens of bacteria, and fungi were also detected. The richness and diversity of airway microbiota and mycobiota were markedly reduced in transplant subjects compared with control subjects, as well as in CF patients with decreased lung function and poor clinical status (Charlson et al., 2012b; Delhaes et al., 2012). In addition, macromycetes which had been found on the common trees in the forest in northern France were identified in sputum samples from several CF patients (Figures 1 and 2). This finding emphasized the significance of the exposure of the patients to the outdoor environment (Delhaes et al., 2012; van Woerden et al., 2013).

Interestingly, the *Malassezia* genus, which was detected as abundant taxa in the CF patients (Delhaes et al., 2012; Willger et al., 2014), was also found in patients with asthma but not in the control group (van Woerden et al., 2013; Figures 1 and 2). This genus, which was recently identified as the dominant taxa on human core body and arms skin (Findley et al., 2013) is known to be associated with atopic dermatitidis. In addition, pyrosequencing studies revealed the presence of *Malassezia* species in oral cavity (Dupuy et al., 2014) and in sinonasal mucosa of patients with chronic rhinosinusitis (Cleland et al., 2014). These results suggest that *Malassezia* species should have a greater interest in lung mycobiome research.

Up to now, lung mycobiome exhibited significant composition differences between patients with CRD and healthy individuals according to the limited studies based on next generation sequencing (NGS) technology (Charlson et al., 2012b; Delhaes et al., 2012; van Woerden et al., 2013; Willger et al., 2014). Moreover, the authors were also able to identify the changes in the lung mycobiota over different time and/or during therapies administered and correlate these changes with the clinical context. Although the sample size was rather small (eight sputum samples from four CF patients were studied), Delhaes et al. (2012) suggested that the respiratory mycobiota plays a role in the development of CF lung pathology. *Candida* was the most commonly genus isolated in CF sputum samples, in particular *C. albicans* (Charlson et al., 2012b; Delhaes et al., 2012; Willger et al., 2014). *C. albicans* has been related to lung function decline in CF (Chotirmall et al., 2010), even if its pathogenic role remained controversial. In addition, close interactions between fungi and bacteria in the lung microbiome of CRD patients have been proposed (Delhaes et al., 2012; Marsland and Gollwitzer, 2014), in agreement with the recent ecological model of CF lungs, named the “climax-attack” community (Conrad et al., 2013). In this model, climax communities are composed of persistent bacterial and fungal populations (for example *Pseudomonas aeruginosa*, *Staphylococcus aureus*, *Aspergillus* spp*.*, *Scedosporium* spp*.*) which colonize the airway during the stable periods. The attack communities are more virulent, usually composed of pathogenic microorganisms which are associated with pulmonary exacerbations such as *S. pneumoniae*, *H. influenza*, *Rhinovirus*, *Adenovirus*, etc.). Both attack and climax communities can coexist in CF patients’ lungs and together contribute to the lung function decline. In this model, the climax community seems to be able to avoid the patients’ immune responses and/or is more resistant to broad-spectrum antibiotic therapy. In contrast, the attack communities are expected to be more susceptible to antibiotics.

This model or other mathematical modeling together with metagenomic methods could help predict the effects of antimicrobial treatments on the lung microbiome, leading to a more effective use of antibiotic and/or antifungal drugs with different treatment regimens. On the whole, these findings provide novel approaches to addressing the relationship between microbial communities and pulmonary disease. It could help to assess lung infections by favoring the development of new therapeutic approaches and models. More investigations should be carried on this field to improve our knowledge of the role of the mycobiome in the lung microbiome and in chronic diseases, such as CRD.

## RELATIONSHIPS BETWEEN THE LUNG AND GUT MICROBIOMES: FOCUS ON THE MYCOBIOME

Despite limited research data, the concept of a relationship between the respiratory and gut microbiomes has recently been considered (Duytschaever et al., 2011; Madan et al., 2012; Huang, 2013). The human gastrointestinal tract is colonized by a complex microbial community, which has been extensively studied during the last decade. The gastrointestinal microbiome is now considered an organ with an important role in digestive metabolism, in the development and homeostasis of host inflammatory immune responses. Dysbiosis of the gut microbiome has been associated with a variety of chronic diseases, such as inflammatory bowel disease, obesity, type 2 diabetes, and asthma (Turnbaugh and Gordon, 2009; Huang, 2013; Karlsson et al., 2013). Furthermore, there is increasing evidence that the gastrointestinal mucosa is a predominant site of microbiome–host interactions and can contribute to the development of immune responses at distal mucosal sites.

Due to ethical considerations, the small size of samples, and/or technical problems, studies conducted on human beings to investigate the relationships between the gut and lung microbiomes are limited. However, animal models have been developed to test the influence of the gut microbiome on the lung microbiome and immunity. Using a mouse model of cefoperazone-induced gastrointestinal microbiome disruption, Noverr et al. (2004, 2005) demonstrated that the gastrointestinal dysbiosis was characterized by increased numbers of enteric bacteria and *C. albicans*. They also proved the impact of the gastrointestinal microbiome perturbation on immune responses in the lung. Only mice with altered gastrointestinal microbiota developed airway allergic responses to intranasal administration of ovalbumin, associated with significant increases in the levels of eosinophils, mast cells, interleukin (IL)-5 and IL-13, INFγ in the lungs, and IgE in serum. The eosinophilic nature of the inflammatory response was confirmed by lung histological analysis. The allergic response was also induced by intranasally delivered conidia of *A. fumigatus* using the same model to perturb the gut microbiome either in Balb/c or C57BL/6 mice. These studies demonstrated that the effects of gastrointestinal microbiome disruption were independent of the host genetic background, and that the airway allergic responses following this dysbiosis were independent of the nature of the allergen challenge but require IL-13 production in mouse models (Noverr et al., 2004, 2005)*.* A more recent study (Barfod et al., 2013) compared the bacterial communities isolated from BAL fluids, lung tissue biopsies, fecal samples, and vaginal lavage fluids of BALB/c mice. It has shown that the lung microbiota is distinct from the cecal microbiota but overlaps with the vaginal microbiota in this animal model. Barfod et al. (2013) recommended taking into account the lung microbiome and mycobiome when studying the pathogenesis of inflammatory lung diseases.

To date, only two clinical trials have been conducted in CRD populations to document the relationships between the gut microbiome and the lungs (Duytschaever et al., 2011; Madan et al., 2012). Twenty-one children with CF and 24 healthy siblings were included in a cross-sectional study (Duytschaever et al., 2011) in an effort to find any significant differences between the relative composition and temporal stability of the predominant fecal microbiome in CF patients and those in their healthy siblings, using culture and DGGE methods. Siblings were chosen as controls for patients with CF to avoid the effects of several factors that may influence the composition of the gastrointestinal microbiota, such as genetic background, age, and environmental factors. However, other factors, including antibiotic management in CF or the effect of daily diet, certainly differed since patients with CF are recommended to consume a high-fat diet to meet their energy needs. While enterobacterial counts were consistently higher in CF patients, no typical DGGE fingerprints were found from the cross-sectional study. The longitudinal study performed on two patient–sibling pairs exhibited a trend toward lower temporal stability and richness in the fecal microbiome of CF patients. Both cross-sectional and longitudinal studies provided primary evidence of a continuous state of intestinal dysbiosis in CF children compared to their siblings. Both the intrinsic characteristics of the disease (such as abnormal mucus secretions and pancreatic insufficiency) and the detrimental effects of intensive antimicrobial treatment courses most likely play key roles in this dysbiosis.

The second study, using the 16 rDNA gene pyrosequencing (Madan et al., 2012), investigated the respiratory and intestinal microbiota development in infants with CF followed from birth to 21 months. In observing the dominant genera, some similar bacteria were found in both gut and respiratory microbiota, such as *Veillonella* and *Streptococcus*. Bacterial diversity increased significantly over time in both the respiratory and intestinal tracts, and in which the respiratory microbiota increased more rapidly. A significant proportion of bacteria increasing in the gut were also increasing in the respiratory tract. Furthermore, changes in diet (such as breast-feeding or introduction of solid foods) also result in modified microbiomes, suggesting an authentic link between nutrition and the development of a microbial lung community (Madan et al., 2012). These data suggest that gut colonization patterns and nutritional factors play key roles in the development of the respiratory microbiome.

Changes in the intestinal microbiome also reflect changes in the oropharyngeal microbiome (including the mycobiome component), which may directly influence the respiratory microbiome and host immune response through microaspiration (Charlson et al., 2012b). The mycobiota of the oral cavity in healthy individuals has been well characterized (Ghannoum et al., 2010; Dupuy et al., 2014). Among the 15 genera identified and composed of cultivable and non-cultivable species, *Candida* [isolated from 75% of all study participants (Ghannoum et al., 2010)], *Aspergillus*, *Fusarium*, and *Cryptococcus* were predominant. More recently, a high prevalence and abundance of the genus *Malassezia* was discovered by revising current practices in sequence-based taxonomy assignments (Dupuy et al., 2014). Like the oral bacterial microbiome, the oral mycobiome exhibited great variation within and between individuals that may influence the respiratory and intestinal mycobiomes in healthy individuals as well as in patients (Noverr et al., 2004, 2005; Aas et al., 2005; Ghannoum et al., 2010; Kieninger et al., 2013; Dupuy et al., 2014; Mukherjee et al., 2014).

Altogether, these studies support the concept that the indigenous microbiome and mycobiome of the gastrointestinal tract have a profound influence on the development and maintenance of the respiratory microbiome, as well as lung immunity and inflammation. Further studies targeting intestinal–respiratory microbiome interactions are likely to yield important insights into the dynamics and homeostasis of microbiomes, consequently yielding a better understanding of how intestinal dysbiosis may have notable functional consequences in the pathogenesis of CRD. These findings will represent opportunities for establishing the clinical relevance of early intervention in CRD with altered dietary or frequent antibiotic therapy that may contribute to the development of dysbiosis, or probiotic strategies that might change the colonization of the gut plus lung and thereby improve patients’ outcomes. As this field advances over the next several years, we anticipate that studies using larger cohorts, control groups such as siblings, multicenter designs, and longitudinal sampling will add to our knowledge of the lung microbiome and mycobiome.

## OUTLOOKS: THE RESPIRATORY MYCOBIOME OR A FORGOTTEN PLAYER THAT DESERVES OUR ATTENTION?

**FIGURE 3:**
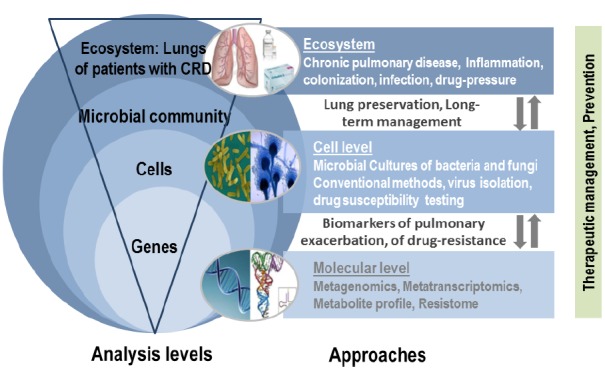
**Integrative research based on the lung mycobiome, virome, and bacteriome**.

Next generation sequencing technologies provide the opportunity to simultaneously analyze the microbial community (bacteria, viruses, and fungi) as a whole in an integrative research methodology without *a priori* knowledge of existing microorganisms, and consequently represent the most promising investigational strategy in the context of CRD (Figure 3). In the last half decade, interest in the human lung bacteriome and its role in developing CRD have increased substantially. As in the intestinal microbiome, bacteria are certainly the most represented microorganisms of the respiratory microbiome, but Archaea, viruses (including phages), and Eukaryota (including fungi) also formed part of this respiratory microbiome, and may reveal clinical significances especially when dysbiosis occurs. However, little to nothing is known about the role of fungi in establishing and maintaining a healthy respiratory ecosystem, or on the other hand in facilitating pulmonary diseases. In agreement with recent published data (Findley et al., 2013; Huffnagle and Noverr, 2013), fungi may play an important role in the stability of the human microbial community, thus affecting human health and disease.

Given the significantly increased rate of allergic diseases such as asthma in the westernized countries, understanding the role of the lung microbiome especially the mycobiome will be of interest in coming decades. In addition, there is evidence that the fungal exposure is associated with asthma and chronic inflammatory pulmonary diseases (Reponen et al., 2011, 2012; Hulin et al., 2013; Kieninger et al., 2013; Pringle, 2013). Fungi are also potential pathogens of the lungs while they probably act as commensals in the gastrointestinal tract, making the mycobiome analysis valuable, or even higher in the respiratory tract.

When considering the respiratory microbiome as a complex and continuous polymicrobial ecosystem composed of viruses, bacteria, and fungi (as a parallel from Island biogeography recently proposed by Whiteson et al. (2014)), these microorganisms may have mutual interferences. Unfortunately, their co-occurrence in the lung microbiome is poorly appreciated. To date, few studies underlined the impact of cross-kingdom synergy between bacteria and fungus in the respiratory tract and the sinonasal mucosa (Delhaes et al., 2012; Boase et al., 2013; Cleland et al., 2014). The limited understanding of fungal–bacterial interactions and their function in respiratory diseases and healthy lungs results primarily from our incomplete knowledge of the respiratory mycobiome. Overall, information obtained from oral cavity and gastrointestinal ecosystems indicates that fungi influence bacterial behavior through different interactions (i.e., positive and negative influences between and among microbiome members; Duran-Pinedo et al., 2011, 2014; Iliev et al., 2012). As in other body sites, the interactions between fungi and bacteria may occur in the lungs at physical and chemical levels. The physical interactions are mainly represented by co-occurrence or co-exclusion phenomena, while the chemical interactions include metabolic needs, quorum-sensing exchanges, and the production of antimicrobial agents. Fungi and bacteria are able to generate biofilm structures which protect bacteria and/or fungi against desiccation, antibiotic diffusion, or immune cell attacks. It results in the development of strains that are multiresistant to antimicrobial agents and able to disseminate. Co-infection by these pathogens forming mixed communities has elevated virulence and resistance, and resilience properties that are significantly different from those of single-species communities (reviewed in Wargo and Hogan, 2006). Animal models have also shown the impact of cross-kingdom synergy between bacteria and fungi, supporting a potentially mutualistic partnership between *Candida* and *Streptococcus* (Roux et al., 2009; Diaz et al., 2012; Mason et al., 2012; Xu et al., 2014).

By comparing cross-kingdom relationships associated with health and CRD using *in vitro* and *in vivo* studies, “omics” approaches will revolutionize the identification and characterization of fungal–bacterial interactions. Correlation network information already allowed authors to identify *Prevotella* species as growth co-partners of *Tannerella* sp. HOT286 and consequently to cultivate the uncultivated *Tannerella* bacteria in a co-culture system (Duran-Pinedo et al., 2011). Other authors revealed significant inter-bacteria associations at different body sites of healthy people (Faust et al., 2012). By exploring co-occurrence patterns of fungi and bacteria, Mukherjee et al. (2014) identified and explored antagonisms between *Candida* and *Pichia* yeasts in oral rinse samples. Furthermore, these bacterial–fungal interactions often have an important impact on the biology of the host metabolism and immunity as demonstrated in the gut microbiome (reviewed in Ianiro et al., 2014; Underhill and Iliev, 2014); they might also play a role in chronic pulmonary diseases and the pulmonary transplantation system, which emphasizes the need to define the respiratory mycobiome.

## CONCLUSION AND FUTURE DIRECTIONS

For a few decades, metagenomic research has focused on the bacterial microbiome; however, clinical and experimental studies have started to highlight a role for fungi in the human microbiome in general, and in the respiratory microbiome in particular. Such a polymicrobial community has emergent properties that cannot be inferred by studying its components separately. In this respect, the “omics” approaches will be significantly helpful in identifying and deciphering candidate fungal–bacterial interactions essential to maintaining a healthy respiratory microbiome, or on the other hand to facilitate CRD.

The rapid development of NGS technologies has opened up possibilities to better define in a novel way, the compositions and functions of the respiratory mycobiome and microbiome in homeostasis, during infection, and in the context of CRD. It is now essential to develop robust universal methodological strategies, and to implement large multicenter studies. In spite of recent methodological advances, the current metagenomic approaches used to study the mycobiome still have certain limitations. As proposed by Diaz et al. (2014), there are numerous pitfalls associated with the mycobiome that we need to challenge collectively. First, fungal cells are notoriously difficult to break open and might require chemical or mechanical lysis as a pre-extraction step (Chen et al., 2002; Fredricks et al., 2005; Griffiths, 2006; Plassart et al., 2012; Dupuy et al., 2014; Goldschmidt et al., 2014). Another technical point in measuring fungi using a DNA based method, is to be able to distinguish the DNA of living microorganisms and that of dead ones. Several recent studies (Nocker and Richter-Heitmann, 2010; Rogers et al., 2013; Chiao et al., 2014; Cuthbertson et al., 2014) demonstrated that a sample pre-treatment with propidium monoazide (a chemical molecule able to penetrate exclusively into cells with an intact membrane which are considered as viable cells) modified the bacterial community profiles obtained by NGS. The second challenge is to improve the taxonomic assignment quality by establishing an accurate updated fungal database. Since the internal transcribed spacer (ITS) regions of the rDNA are widely used for fungal species identification and recently formalized as universal DNA barcode markers for fungi (Delhaes et al., 2012; Schoch et al., 2012), several projects have been conducted to develop fungal databases, such as ITSDB or UNITE for QIIME released^3^ (Diaz et al., 2014) or ISHAM ITS Database^4^. The later ITS database was recently generated from quality controlled ITS sequences, which represent the actual sequence variation found in each species. Given that fungi represent the largest family of microorganisms with multiple names, another challenge is to prevent unnecessary nomenclatural flux, as recently proposed (de Hoog et al., 2014). In this context, the ISHAM ITS or UNITE databases represent a nice opportunity to create an accurate database with consensual nomenclature. Further advances in data generation and analysis are also required to minimize errors and misinterpretations, to make cross-studies and multicenter trials more feasible, and finally to link the bench and the clinic.

To understand the biological function of the respiratory mycobiome in host inflammatory lung responses, its implication in CRD progression, its role in local cross-kingdom microbial interactions, as well as in the cross-talk between the intestinal and lung microbiomes, future large-scale cross-sectional and longitudinal studies must be conducted to answer these questions raised. By analogy to the microbiota signatures recently proposed for assessing responses to dietary interventions in obese individuals (Korpela et al., 2014) and for predicting future exacerbations in bronchiectasis (Rogers et al., 2014), determining both specific mycobiota and microbiota respiratory signatures could be useful for prophylactic or therapeutic management in CRD. These microbiome and mycobiome signatures might also serve as specific biomarkers preceding the clinical manifestations of disease (CRD), which should be more sensitive than routine culture methods. They might then be an indicator for timely clinical intervention and successful disease management (Lim et al., 2014). The goal is to establish both the mycobiome and microbiome in the respiratory tract over time during a respiratory superinfection or an acute pulmonary exacerbation, in order to provide key elements (signatures, dysbiosis) for early diagnosis and appropriate preventive therapy of secondary infections. Longitudinal studies will help in analyzing fungal–bacterial interactions simultaneously during the CRD progression and therapy outcome. All these additional studies are needed to generate hypotheses that in vitro and animal models will explore.

By taking into account the total respiratory microbiome (that does not only consist of bacteria but also fungi, phages and viruses), these future studies will be dramatically instrumental in improving our knowledge of the pathogenesis of CRD and in developing innovative therapies.

### Conflict of Interest Statement

The authors declare that the research was conducted in the absence of any commercial or financial relationships that could be construed as a potential conflict of interest.

## Footnotes

1 http://www.hmpdacc.org/

2 http://www.metahit.eu/

3 http://qiime.org/home_static/dataFiles.html

4 http://its.mycologylab.org/

## References

[B1] Aas J. A. Paster B. J. Stokes L. N. Olsen I. Dewhirst F. E. (2005). Defining the normal bacterial flora of the oral cavity. J. Clin. Microbiol. 43, 5721–5732. 10.1128/JCM.43.11.5721-5732.2005 16272510 PMC1287824

[B2] Amin R. Dupuis A. Aaron S. D. Ratjen F. (2010). The effect of chronic infection with *Aspergillus fumigatus* on lung function and hospitalization in patients with cystic fibrosis. Chest 137, 171–176. 10.1378/chest.09-1103 19567494

[B3] Armstead J. Morris J. Denning D. W. (2014). Multi-country estimate of different manifestations of aspergillosis in cystic fibrosis. PLoS ONE 9:e98502. 10.1371/journal.pone.0098502 24914809 PMC4051580

[B4] Barfod K. Roggenbuck M. Hansen L. Schjørring S. Larsen S. Sørensen S. (2013). The murine lung microbiome in relation to the intestinal and vaginal bacterial communities. BMC Microbiol. 13:303. 10.1186/1471-2180-13-303 24373613 PMC3878784

[B5] Bittar F. Richet H. Dubus J.-C. Reynaud-Gaubert M. Stremler N. Sarles J. (2008). Molecular detection of multiple emerging pathogens in sputa from cystic fibrosis patients. PLoS ONE 3:e2908. 10.1371/journal.pone.0002908 18682840 PMC2483419

[B6] Boase S. Foreman A. Cleland E. Tan L. Melton-Kreft R. Pant H. (2013). The microbiome of chronic rhinosinusitis: culture, molecular diagnostics and biofilm detection. BMC Infect. Dis. 13:210. 10.1186/1471-2334-13-210 23656607 PMC3654890

[B7] Bousquet A. Malfuson J.-V. Sanmartin N. Konopacki J. MacNab C. Souleau B. (2014). An 8-year survey of strains identified in blood cultures in a clinical haematology unit. Clin. Microbiol. Infect. 20, O7–O12. 10.1111/1469-0691.12294 23826912

[B8] Carmody L. A. Zhao J. Schloss P. D. Petrosino J. F. Murray S. Young V. B. (2013). Changes in cystic fibrosis airway microbiota at pulmonary exacerbation. Ann. Am. Thorac. Soc. 10, 179–187. 10.1513/AnnalsATS.201211-107OC 23802813 PMC3960905

[B9] Chabé M. Aliouat-Denis C.-M. Delhaes L. Aliouat E. M. Viscogliosi E. Dei-Cas E. (2011). Pneumocystis: from a doubtful unique entity to a group of highly diversified fungal species. FEMS Yeast Res. 11, 2–17. 10.1111/j.1567-1364.2010.00698.x 21114625

[B10] Charlson E. S. Bittinger K. Chen J. Diamond J. M. Li H. Collman R. G. (2012a). Assessing bacterial populations in the lung by replicate analysis of samples from the upper and lower respiratory tracts. PLoS ONE 7:e42786. 10.1371/journal.pone.0042786 22970118 PMC3435383

[B11] Charlson E. S. Diamond J. M. Bittinger K. Fitzgerald A. S. Yadav A. Haas A. R. (2012b). Lung-enriched organisms and aberrant bacterial and fungal respiratory microbiota after lung transplant. Am. J. Respir. Crit. Care Med. 186, 536–545. 10.1164/rccm.201204-0693OC 22798321 PMC3480531

[B12] Charlson E. S. Chen J. Custers-Allen R. Bittinger K. Li H. Sinha R. (2010). Disordered microbial communities in the upper respiratory tract of cigarette smokers. PLoS ONE 5:e15216. 10.1371/journal.pone.0015216 21188149 PMC3004851

[B13] Chen S. C. A. Halliday C. L. Meyer W. (2002). A review of nucleic acid-based diagnostic tests for systemic mycoses with an emphasis on polymerase chain reaction-based assays. Med. Mycol. 40, 333–357 12230214 10.1080/mmy.40.4.333.357

[B14] Chen T.-C. Chen Y.-H. Chen Y.-C. Lu P.-L. (2012). Fluconazole exposure rather than clonal spreading is correlated with the emergence of *Candida glabrata* with cross-resistance to triazole antifungal agents. Kaohsiung J. Med. Sci. 28, 306–315. 10.1016/j.kjms.2011.11.011 22632885 PMC11916436

[B15] Chiao T. Clancy T. M. Pinto A. Xi C. Raskin L. (2014). Differential resistance of drinking water bacterial populations to monochloramine disinfection. Environ. Sci. Technol. 48, 4038–4047. 10.1021/es4055725 24625288

[B16] Chotirmall S. H. O’Donoghue E. Bennett K. Gunaratnam C. O’Neill S. J. McElvaney N. G. (2010). Sputum *Candida albicans* presages FEV decline and hospital-treated exacerbations in cystic fibrosis. Chest 138, 1186–1195. 10.1378/chest.09-2996 20472859

[B17] Cleland E. J. Bassioni A. Boase S. Dowd S. Vreugde S. Wormald P.-J. (2014). The fungal microbiome in chronic rhinosinusitis: richness, diversity, postoperative changes and patient outcomes: fungal microbiome in CRS. Int. Forum Allergy Rhinol. 4, 259–265. 10.1002/alr.21297 24500871

[B18] Conrad D. Haynes M. Salamon P. Rainey P. B. Youle M. Rohwer F. (2013). Cystic fibrosis therapy: a community ecology perspective. Am. J. Respir. Cell Mol. Biol. 48, 150–156. 10.1165/rcmb.2012-0059PS 23103995 PMC3604065

[B19] Cui L. Morris A. Ghedin E. (2013). The human mycobiome in health and disease. Genome Med. 5, 63. 10.1186/gm467 23899327 PMC3978422

[B20] Cuthbertson L. Rogers G. B. Walker A. W. Oliver A. Hoffman L. R. Carroll M. P. (2014). Implications of multiple freeze-thawing on respiratory samples for culture-independent analyses. J. Cyst. Fibros. 4–7 10.1016/j.jcf.2014.10.004 [Epub ahead of print] PMC4793934 25459563

[B21] Dawood F. S. Chaves S. S. Perez A. Reingold A. Meek J. Farley M. M. (2014). complications and associated bacterial coinfections among children hospitalized with seasonal or pandemic influenza, United States, 2003–2010. J. Infect. Dis. 209, 686–694. 10.1093/infdis/jit473 23986545

[B22] de Hoog G. S. Chaturvedi V. Denning D. W. Dyer P. S. Frisvad J. C. Geiser D. (2014). Name changes in medically important fungi and their implications for clinical practice. J. Clin. Microbiol. 10.1128/JCM.02016-14 PMC4365198 25297326

[B23] Delhaes L. Monchy S. Fréalle E. Hubans C. Salleron J. Leroy S. (2012). The airway microbiota in cystic fibrosis: a complex fungal and bacterial community—implications for therapeutic management. PLoS ONE 7:e36313 10.1371/journal.pone.0036313 22558432 PMC3338676

[B24] Denning D. W. Pashley C. Hartl D. Wardlaw A. Godet C. Del Giacco S. (2014). Fungal allergy in asthma-state of the art and research needs. Clin. Transl. Allergy 4, 14. 10.1186/2045-7022-4-14 24735832 PMC4005466

[B25] Diaz P. I. Strausbaugh L. D. Dongari-Bagtzoglou A. (2014). Fungal–bacterial interactions and their relevance to oral health: linking the clinic and the bench. Front. Cell. Infect. Microbiol. 4:101. 10.3389/fcimb.2014.00101 25120959 PMC4114182

[B26] Diaz P. I. Xie Z. Sobue T. Thompson A. Biyikoglu B. Ricker A. (2012). Synergistic interaction between *Candida albicans* and commensal oral streptococci in a novel in vitro mucosal model. Infect. Immun. 80, 620–632. 10.1128/IAI.05896-11 22104105 PMC3264323

[B27] Dupuy A. K. David M. S. Li L. Heider T. N. Peterson J. D. Montano E. A. (2014). Redefining the human oral mycobiome with improved practices in amplicon-based taxonomy: discovery of malassezia as a prominent commensal. PLoS ONE 9:e90899. 10.1371/journal.pone.0090899 24614173 PMC3948697

[B28] Duran-Pinedo A. E. Chen T. Teles R. Starr J. R. Wang X. Krishnan K. (2014). Community-wide transcriptome of the oral microbiome in subjects with and without periodontitis. ISME J. 8, 1659–1672. 10.1038/ismej.2014.23 24599074 PMC4817619

[B29] Duran-Pinedo A. E. Paster B. Teles R. Frias-Lopez J. (2011). Correlation network analysis applied to complex biofilm communities. PLoS ONE 6:e28438. 10.1371/journal.pone.0028438 22163302 PMC3233593

[B30] Duytschaever G. Huys G. Bekaert M. Boulanger L. De Boeck K. Vandamme P. (2011). Cross-sectional and longitudinal comparisons of the predominant fecal microbiota compositions of a group of pediatric patients with cystic fibrosis and their healthy siblings. Appl. Environ. Microbiol. 77, 8015–8024. 10.1128/AEM.05933-11 21926193 PMC3208981

[B31] Erb-Downward J. R. Thompson D. L. Han M. K. Freeman C. M. McCloskey L. Schmidt L. A. (2011). Analysis of the lung microbiome in the “healthy” smoker and in COPD. PLoS ONE 6:e16384. 10.1371/journal.pone.0016384 21364979 PMC3043049

[B32] Faust K. Sathirapongsasuti J. F. Izard J. Segata N. Gevers D. Raes J. (2012). Microbial co-occurrence relationships in the human microbiome. PLoS Comput. Biol. 8:e1002606. 10.1371/journal.pcbi.1002606 22807668 PMC3395616

[B33] Filkins L. M. Hampton T. H. Gifford A. H. Gross M. J. Hogan D. A. Sogin M. L. (2012). Prevalence of streptococci and increased polymicrobial diversity associated with cystic fibrosis patient stability. J. Bacteriol. 194, 4709–4717. 10.1128/JB.00566-12 22753064 PMC3415522

[B34] Fillaux J. Brémont F. Murris M. Cassaing S. Rittié J.-L. Tétu L. (2012). Assessment of Aspergillus sensitization or persistent carriage as a factor in lung function impairment in cystic fibrosis patients. Scand. J. Infect. Dis. 44, 842–847. 10.3109/00365548.2012.695454 22831545

[B35] Findley K. Oh J. Yang J. Conlan S. Deming C. Meyer J. A. (2013). Topographic diversity of fungal and bacterial communities in human skin. Nature 498, 367–370. 10.1038/nature12171 23698366 PMC3711185

[B36] Fisher M. C. Henk D. A. Briggs C. J. Brownstein J. S. Madoff L. C. McCraw S. L. (2012). Emerging fungal threats to animal, plant and ecosystem health. Nature 484, 186–194. 10.1038/nature10947 22498624 PMC3821985

[B37] Fitzpatrick M. E. Sethi S. Daley C. L. Ray P. Beck J. M. Gingo M. R. (2014). Infections in “noninfectious” lung diseases. Ann. Am. Thorac. Soc. 11, S221–S226. 10.1513/AnnalsATS.201401-041PL 25148428 PMC4200575

[B38] Fodor A. A. Klem E. R. Gilpin D. F. Elborn J. S. Boucher R. C. Tunney M. M. (2012). The adult cystic fibrosis airway microbiota is stable over time and infection type, and highly resilient to antibiotic treatment of exacerbations. PLoS ONE 7:e45001. 10.1371/journal.pone.0045001 23049765 PMC3458854

[B39] Fredricks D. N. Smith C. Meier A. (2005). Comparison of six DNA extraction methods for recovery of fungal DNA as assessed by quantitative PCR. J. Clin. Microbiol. 43, 5122–5128. 10.1128/JCM.43.10.5122-5128.2005 16207973 PMC1248488

[B40] Ghannoum M. A. Jurevic R. J. Mukherjee P. K. Cui F. Sikaroodi M. Naqvi A. (2010). Characterization of the oral fungal microbiome (Mycobiome) in healthy individuals. PLoS Pathog. 6:e1000713. 10.1371/journal.ppat.1000713 20072605 PMC2795202

[B41] Goffard A. Lambert V. Salleron J. Herwegh S. Engelmann I. Pinel C. (2014). Virus and cystic fibrosis: rhinoviruses are associated with exacerbations in adult patients. J. Clin. Virol. 60, 147–153. 10.1016/j.jcv.2014.02.005 24637203 PMC7108260

[B42] Goldschmidt P. Degorge S. Merabet L. Chaumeil C. (2014). Enzymatic treatment of specimens before dna extraction directly influences molecular detection of infectious agents. PLoS ONE 9:e94886. 10.1371/journal.pone.0094886 24936792 PMC4061000

[B43] Griffiths L. J. (2006). Comparison of DNA extraction methods for *Aspergillus fumigatus* using real-time PCR. J. Med. Microbiol. 55, 1187–1191. 10.1099/jmm.0.46510-0 16914647

[B44] Harrison M. J. Twomey K. B. McCarthy Y. O’Connell O. J. Alston M. Febrer M. (2013). The role of second-generation sequencing in describing the fungal microbiota in the adult cystic fibrosis (CF) airway and its correlation with clinical phenotype. J. Cyst. Fibros. 12, S16 10.1016/S1569-1993(13)60046-6

[B45] Hooper L. V. (2001). Molecular analysis of commensal host–microbial relationships in the intestine. Science 291, 881–884. 10.1126/science.291.5505.881 11157169

[B46] Horré R. Symoens F. Delhaes L. Bouchara J.-P. (2010). Fungal respiratory infections in cystic fibrosis: a growing problem. Med. Mycol. 48, S1–S3. 10.3109/13693786.2010.529304 21067320

[B47] Huang Y. J. (2013). Asthma microbiome studies and the potential for new therapeutic strategies. Curr. Allergy Asthma Rep. 13, 453–461. 10.1007/s11882-013-0355-y 23709178 PMC3778058

[B48] Huang Y. J. Charlson E. S. Collman R. G. Colombini-Hatch S. Martinez F. D. Senior R. M. (2013). The role of the lung microbiome in health and disease. a national heart, lung, and blood institute workshop report. Am. J. Respir. Crit. Care Med. 187, 1382–1387. 10.1164/rccm.201303-0488WS 23614695 PMC5155250

[B49] Huffnagle G. B. Noverr M. C. (2013). The emerging world of the fungal microbiome. Trends Microbiol. 21, 334–341. 10.1016/j.tim.2013.04.002 23685069 PMC3708484

[B50] Hulin M. Moularat S. Kirchner S. Robine E. Mandin C. Annesi-Maesano I. (2013). Positive associations between respiratory outcomes and fungal index in rural inhabitants of a representative sample of French dwellings. Int. J. Hyg. Environ. Health 216, 155–162. 10.1016/j.ijheh.2012.02.011 22465486

[B51] Huttenhower C. Gevers D. Knight R. Abubucker S. Badger J. H. Chinwalla A. T. (2012). Structure, function and diversity of the healthy human microbiome. Nature 486, 207–214. 10.1038/nature11234 22699609 PMC3564958

[B52] Ianiro G. Bruno G. Lopetuso L. Beghella F. B. Laterza L. D’Aversa F. (2014). Role of yeasts in healthy and impaired gut microbiota: the gut mycome. Curr. Pharm. Des. 20, 4565–4569. 24180411 10.2174/13816128113196660723

[B53] Iliev I. D. Funari V. A. Taylor K. D. Nguyen Q. Reyes C. N. Strom S. P. (2012). Interactions between commensal fungi and the C-type lectin receptor dectin-1 influence colitis. Science 336, 1314–1317. 10.1126/science.1221789 22674328 PMC3432565

[B54] Karlsson F. H. Tremaroli V. Nookaew I. Bergström G. Behre C. J. Fagerberg B. (2013). Gut metagenome in European women with normal, impaired and diabetic glucose control. Nature 498, 99–103. 10.1038/nature12198 23719380

[B55] Kieninger E. Singer F. Tapparel C. Alves M. P. Latzin P. Tan H.-L. (2013). High rhinovirus burden in lower airways of children with cystic fibrosis. Chest 143, 782–790. 10.1378/chest.12-0954 23188200

[B56] Knutsen A. P. Bush R. K. Demain J. G. Denning D. W. Dixit A. Fairs A. (2012). Fungi and allergic lower respiratory tract diseases. J. Allergy Clin. Immunol. 129, 280–291. 10.1016/j.jaci.2011.12.970 22284927

[B57] Korpela K. Flint H. J. Johnstone A. M. Lappi J. Poutanen K. Dewulf E. (2014). Gut microbiota signatures predict host and microbiota responses to dietary interventions in obese individuals. PLoS ONE 9:e90702. 10.1371/journal.pone.0090702 24603757 PMC3946202

[B58] Lim Y. W. Evangelista J. S. Schmieder R. Bailey B. Haynes M. Furlan M. (2014). Clinical insights from metagenomic analysis of sputum samples from patients with cystic fibrosis. J. Clin. Microbiol. 52, 425–437. 10.1128/JCM.02204-13 24478471 PMC3911355

[B59] Lim Y. W. Schmieder R. Haynes M. Willner D. Furlan M. Youle M. (2013). Metagenomics and metatranscriptomics: windows on CF-associated viral and microbial communities. J. Cyst. Fibros. 12, 154–164. 10.1016/j.jcf.2012.07.009 22951208 PMC3534838

[B60] Lu Q. van den Ende A. H. de Hoog G. S. Li R. Accoceberry I. Durand-Joly I. (2011). Reverse line blot hybridisation screening of *Pseudallescheria*/*Scedosporium* species in patients with cystic fibrosis: RLB of *Scedosporium* spp. in cystic fibrosis. Mycoses 54, 5–11. 10.1111/j.1439-0507.2011.02108.x 21995657

[B61] Madan J. C. Koestler D. C. Stanton B. A. Davidson L. Moulton L. A. Housman M. L. (2012). Serial analysis of the gut and respiratory microbiome in cystic fibrosis in infancy: interaction between intestinal and respiratory tracts and impact of nutritional exposures. mBio 3, e00251–12–e00251–12 10.1128/mBio.00251-12 22911969 PMC3428694

[B62] Marguet C. Favennec L. Matray O. Bertout S. Giraud S. Couderc L. (2012). Clinical and microbiological efficacy of micafungin on *Geosmithia argillacea* infection in a cystic fibrosis patient. Med. Mycol. Case Rep. 1, 79–81. 10.1016/j.mmcr.2012.08.004 24371745 PMC3854624

[B63] Marsland B. J. Gollwitzer E. S. (2014). Host–microorganism interactions in lung diseases. Nat. Rev. Immunol. 14, 827–835. 10.1038/nri3769 25421702

[B64] Mason K. L. Erb Downward J. R. Falkowski N. R. Young V. B. Kao J. Y. Huffnagle G. B. (2012). Interplay between the gastric bacterial microbiota and *Candida albicans* during postantibiotic recolonization and gastritis. Infect. Immun. 80, 150–158. 10.1128/IAI.05162-11 21986629 PMC3255670

[B65] McCullers J. A. (2014). The co-pathogenesis of influenza viruses with bacteria in the lung. Nat. Rev. Microbiol. 12, 252–262. 10.1038/nrmicro3231 24590244

[B66] Mounier J. Gouëllo A. Keravec M. Le Gal S. Pacini G. Debaets S. (2014). Use of denaturing high-performance liquid chromatography (DHPLC) to characterize the bacterial and fungal airway microbiota of cystic fibrosis patients. J. Microbiol. 52, 307–314. 10.1007/s12275-014-3425-5 24535743

[B67] Mukherjee P. K. Chandra J. Retuerto M. Sikaroodi M. Brown R. E. Jurevic R. (2014). Oral mycobiome analysis of HIV-infected patients: identification of Pichia as an antagonist of opportunistic fungi. PLoS Pathog. 10:e1003996. 10.1371/journal.ppat.1003996 24626467 PMC3953492

[B68] Nocker A. Richter-Heitmann T. (2010). Discrimination between live and dead cells in bacterial communities from environmental water samples analyzed by 454 pyrosequencing. Int. Microbiol. 13, 59–65. 10.2436/20.1501.01.111 20890840

[B69] Noverr M. C. Falkowski N. R. McDonald R. A. Mc Kenzy A. N. Huffnagle G. B. (2005). Development of allergic airway disease in mice following antibiotic therapy and fungal microbiota increase: role of host genetics, antigen and interleukin-13. Infect. Immun. 73, 30–38. 15618138 10.1128/IAI.73.1.30-38.2005 PMC538952

[B70] Noverr M. C. Noggle R. M. Toews G. B. Huffnagle G. B. (2004). Role of antibiotics and fungal microbiota in driving pulmonary allergic responses. Infect. Immun. 72, 4996–5003. 10.1128/IAI.72.9.4996-5003.2004 15321991 PMC517468

[B71] Orgiazzi A. Bianciotto V. Bonfante P. Daghino S. Ghignone S. Lazzari A. (2013). 454 Pyrosequencing analysis of fungal assemblages from geographically distant, disparate soils reveals spatial patterning and a core mycobiome. Diversity 5, 73–98 10.3390/d5010073

[B72] Paniz-Mondolfi A. Talhari C. Sander Hoffmann L. Connor D. L. Talhari S. Bermudez-Villapol L. (2012). Lobomycosis: an emerging disease in humans and delphinidae. Mycoses 55, 298–309. 10.1111/j.1439-0507.2012.02184.x 22429689

[B73] Pashley C. H. Fairs A. Free R. C. Wardlaw A. J. (2012). DNA analysis of outdoor air reveals a high degree of fungal diversity, temporal variability, and genera not seen by spore morphology. Fungal Biol. 116, 214–224. 10.1016/j.funbio.2011.11.004 22289767

[B74] Plassart P. Terrat S. Thomson B. Griffiths R. Dequiedt S. Lelievre M. (2012). Evaluation of the ISO Standard 11063 DNA extraction procedure for assessing soil microbial abundance and community structure. PLoS ONE 7:e44279. 10.1371/journal.pone.0044279 22984486 PMC3439486

[B75] Pringle A. (2013). Asthma and the diversity of fungal spores in air. PLoS Pathog. 9:e1003371. 10.1371/journal.ppat.1003371 23762024 PMC3675135

[B76] Purcell P. Jary H. Perry A. Perry J. D. Stewart C. J. Nelson A. (2014). Polymicrobial airway bacterial communities in adult bronchiectasis patients. BMC Microbiol. 14:130. 10.1186/1471-2180-14-130 24886473 PMC4031157

[B77] Reponen T. Lockey J. Bernstein D. I. Vesper S. J. Levin L. Khurana Hershey G. K. (2012). Infant origins of childhood asthma associated with specific molds. J. Allergy Clin. Immunol. 130, 639–644.e5. 10.1016/j.jaci.2012.05.030 22789397 PMC3432137

[B78] Reponen T. Vesper S. Levin L. Johansson E. Ryan P. Burkle J. (2011). High environmental relative moldiness index during infancy as a predictor of asthma at 7 years of age. Ann. Allergy Asthma Immunol. 107, 120–126. 10.1016/j.anai.2011.04.018 21802019 PMC11610244

[B79] Rogers G. B. Cuthbertson L. Hoffman L. R. Wing P. A. Pope C. Hooftman D. A. (2013). Reducing bias in bacterial community analysis of lower respiratory infections. ISME J. 7, 697–706. 10.1038/ismej.2012.145 23190732 PMC3603400

[B80] Rogers G. B. Zain N. M. M. Bruce K. D. Burr L. D. Chen A. C. Rivett D. W. (2014). A novel microbiota stratification system predicts future exacerbations in bronchiectasis. Ann. Am. Thorac. Soc. 11, 496–503. 10.1513/AnnalsATS.201310-335OC 24592925

[B81] Roux D. Gaudry S. Dreyfuss D. El-Benna J. de Prost N. Denamur E. (2009). *Candida albicans* impairs macrophage function and facilitates *Pseudomonas aeruginosa* pneumonia in rat. Crit. Care Med. 37, 1062–1067. 10.1097/CCM.0b013e31819629d2 19237918

[B82] Schoch C. L. Seifert K. A. Huhndorf S. Robert V. Spouge J. L. Levesque C. A. (2012). Nuclear ribosomal internal transcribed spacer (ITS) region as a universal DNA barcode marker for fungi. Proc. Natl. Acad. Sci. U.S.A. 109, 6241–6246. 10.1073/pnas.1117018109 22454494 PMC3341068

[B83] Speirs J. J. van der Ent C. K. Beekman J. M. (2012). Effects of *Aspergillus fumigatus* colonization on lung function in cystic fibrosis. Curr. Opin. Pulm. Med. 18, 632–638. 10.1097/MCP.0b013e328358d50b 22965276

[B84] The NIH HMP Working Group Peterson J. Garges S. Giovanni M. McInnes P. Wang L. Schloss J. A. (2009). The NIH Human Microbiome Project. Genome Res. 19, 2317–2323. 10.1101/gr.096651.109 19819907 PMC2792171

[B85] Turnbaugh P. J. Gordon J. I. (2009). The core gut microbiome, energy balance and obesity. J. Physiol. 587, 4153–4158. 10.1113/jphysiol.2009.174136 19491241 PMC2754355

[B86] Underhill D. M. Iliev I. D. (2014). The mycobiota: interactions between commensal fungi and the host immune system. Nat. Rev. Immunol. 14, 405–416. 10.1038/nri3684 24854590 PMC4332855

[B87] Vermeulen E. Lagrou K. Verweij P. E. (2013). Azole resistance in *Aspergillus fumigatus*: a growing public health concern. Curr. Opin. Infect. Dis. 26, 493–500. 10.1097/QCO.0000000000000005 24126719

[B88] Wargo M. J. Hogan D. A. (2006). Fungal–bacterial interactions: a mixed bag of mingling microbes. Curr. Opin. Microbiol. 9, 359–364. 10.1016/j.mib.2006.06.001 16777473

[B89] Whiteson K. L. Bailey B. Bergkessel M. Conrad D. Delhaes L. Felts B. (2014). The upper respiratory tract as a microbial source for pulmonary infections in cystic fibrosis. parallels from island biogeography. Am. J. Respir. Crit. Care Med. 189, 1309–1315. 10.1164/rccm.201312-2129PP 24702670 PMC4098084

[B90] Willger S. D. Grim S. L. Dolben E. L. Shipunova A. Hampton T. H. Morrison H. G. (2014). Characterization and quantification of the fungal microbiome in serial samples from individuals with cystic fibrosis. Microbiome 2, 40. 10.1186/2049-2618-2-40 25408892 PMC4236224

[B91] Willner D. Furlan M. Schmieder R. Grasis J. A. Pride D. T. Relman D. A. (2011). Metagenomic detection of phage-encoded platelet-binding factors in the human oral cavity. Proc. Natl. Acad. Sci. U.S.A. 108, 4547–4553. 10.1073/pnas.1000089107 20547834 PMC3063595

[B92] Willner D. Haynes M. R. Furlan M. Hanson N. Kirby B. Lim Y. W. (2012). Case studies of the spatial heterogeneity of DNA viruses in the cystic fibrosis lung. Am. J. Respir. Cell Mol. Biol. 46, 127–131. 10.1165/rcmb.2011-0253OC 21980056 PMC3361360

[B93] van Woerden H. C. Gregory C. Brown R. Marchesi J. R. Hoogendoorn B. Matthews I. P. (2013). Differences in fungi present in induced sputum samples from asthma patients and non-atopic controls: a community based case control study. BMC Infect. Dis. 13:69. 10.1186/1471-2334-13-69 23384395 PMC3570489

[B94] Wurzel D. F. Mackay I. M. Marchant J. M. Wang C. Y. T. Yerkovich S. T. Upham J. W. (2014). Adenovirus species c is associated with chronic suppurative lung diseases in children. Clin. Infect. Dis. 10.1093/cid/ciu225 24748519 PMC4305137

[B95] Xu H. Sobue T. Thompson A. Xie Z. Poon K. Ricker A. (2014). Streptococcal co-infection augments *Candida* pathogenicity by amplifying the mucosal inflammatory response: *Candida*-streptococcal synergy in oral thrush. Cell. Microbiol. 16, 214–231. 10.1111/cmi.12216 24079976 PMC3956708

